# Environmental Cadmium Exposure Perturbs Gut Microbial Dysbiosis in Ducks

**DOI:** 10.3390/vetsci10110649

**Published:** 2023-11-09

**Authors:** Xuefei Wang, Junxian Mi, Kun Yang, Lian Wang

**Affiliations:** Department of Medical Engineering, Henan University of Animal Husbandry and Economy, Zhengzhou 450046, China; 80886@hnuahe.edu.cn (X.W.); 80361@hnuahe.edu.cn (J.M.); 81904@hnuahe.edu.cn (K.Y.)

**Keywords:** cadmium, contaminant, duck, gut microbiota, diversity

## Abstract

**Simple Summary:**

Cadmium is a prevalent heavy metal known to contribute to environmental contamination. Moreover, cadmium in the environment may accumulate in animals and plants and then enter the human body through the food chain, seriously threatening food safety and public health. Currently, cadmium exposure has been demonstrated to cause liver and kidney damage, as well as abnormal bone development, in ducks. However, there is limited research on the impact of cadmium on the gut microbiota of ducks. In this study, we investigated the alterations in the gut microbiota of ducks exposed to cadmium and identified the detrimental effects of cadmium on the gut microbiota.

**Abstract:**

Ore extraction, chemical production, and agricultural fertilizers may release significant amounts of heavy metals, which may eventually accumulate widely in the environment and organisms over time, causing global ecological and health problems. As a recognized environmental contaminant, cadmium has been demonstrated to cause osteoporosis and renal injury, but research regarding the effects of cadmium on gut microbiota in ducks remains scarce. Herein, we aimed to characterize the adverse effects of cadmium on gut microbiota in ducks. Results indicated that cadmium exposure dramatically decreased gut microbial alpha diversity and caused significant changes in the main component of gut microbiota. Moreover, we also observed significant changes in the gut microbial composition in ducks exposed to cadmium. A microbial taxonomic investigation showed that *Firmicutes*, *Bacteroidota*, and *Proteobacteria* were the most preponderant phyla in ducks regardless of treatment, but the compositions and abundances of dominant genera were different. Meanwhile, a Metastats analysis indicated that cadmium exposure also caused a distinct increase in the levels of 1 phylum and 22 genera, as well as a significant reduction in the levels of 1 phylum and 36 genera. In summary, this investigation demonstrated that cadmium exposure could disturb gut microbial homeostasis by decreasing microbial diversity and altering microbial composition. Additionally, under the background of the rising environmental pollution caused by heavy metals, this investigation provides a crucial message for the assessment of environmental risks associated with cadmium exposure.

## 1. Introduction

Gut microbiota has garnered significant interest from microbiologists, biologists, and clinicians due to its crucial role in maintaining host health. The gastrointestinal system is inhabited by a diverse array of microbial communities, including bacteria, fungi, archaea, and viruses [1]. Furthermore, intestinal dysbiosis is linked to inflammation in the intestines, resulting in a compromised intestinal barrier [2]. Early investigations revealed that gut microbiota establishes a mutually beneficial relationship with the host and contributes to multiple physiological functions, such as fat metabolism, digestion, nutrient absorption, and energy utilization [3,4]. Additionally, gut microbiota has also been demonstrated to be closely associated with intestinal barrier, immune regulation, and bone development [5,6]. The correlation between gut microbiota and host diseases has been increasingly uncovered as a result of advancements in high-throughput sequencing technology. Research has shown that the imbalance in gut microbial composition, known as gut microbial dysbiosis, plays a significant role in the development of several gastrointestinal diseases, including inflammatory bowel disease, diarrhea, and constipation [7,8]. Moreover, these microbes, although primarily found in the gastrointestinal tract, can also penetrate the intestinal barrier during gut microbial dysbiosis, which further contributes to the progression of other diseases, such as liver, kidney, and cardiovascular diseases [9,10,11]. The interaction between the gut microbiota and host is a prerequisite for maintaining the host’s health. Therefore, it is highly significant to maintain gut microbial homeostasis. However, this homeostasis is easily disrupted by multiple factors, including diet, age, sex, antibiotics, and environmental pollutants [12,13,14]. Among these factors, environmental pollutants, like heavy metals, are recognized as major contributors to gut microbial dysbiosis [15,16].

In recent decades, the rise of modern industry and increased human activities have led to the discharge of industrial wastewater and wastes containing high levels of heavy-metal pollutants into lakes and land [17,18]. This has become a major cause of global environmental pollution, resulting in substantial economic and medical burdens. According to statistics, millions of tons of toxic heavy metals, such as arsenic, cadmium, copper, mercury, lead, and nickel, are discharged into the environment worldwide each year [19,20]. This discharge is also continuously increasing, resulting in severe environmental pollution. Moreover, it leads to elevated levels of heavy metals in water and soil, posing a significant threat to the health and productivity of animals and plants [19,20]. Notably, heavy metals present in water and soil can enter plants through the root system and subsequently accumulate along the food chain, ultimately endangering human health [21,22].

Cadmium, a non-essential heavy metal, has been identified as a primary carcinogen with a biological half-life of 10–30 years [23]. Industrial activities, such as cadmium mining, refining, and the production of batteries, plastics, paints, and electronics, are the main sources of cadmium in the environment [24,25]. Cadmium contamination has become an inevitable problem in the ecosystem. Statistics show that approximately 13,000 tons of cadmium are produced worldwide each year, some of which cannot be recycled or degraded, leading to its accumulation in the environment [19,20]. Remarkably, this residual cadmium may enter animals and humans through the food chain, resulting in the accumulation of cadmium in the ecosystem and in the body [26,27]. Cadmium can accumulate in multiple organs, such as bones, liver, and brain, causing bone metabolic disease, kidney disease, reproductive disease, and even cancer [28]. Research has demonstrated that poultry are highly susceptible to cadmium toxicity, leading to severe bone metabolic disease and economic losses in the poultry industry. For instance, Ma et al. found that cadmium can cause osteoporosis in ducks by affecting the differentiation of osteoblasts and osteoclasts [19]. Similarly, Wang et al. discovered that cadmium can induce oxidative stress and apoptosis in duck renal tubular epithelial cells [29]. The intestinal tract serves as a significant pathway for the entry of different environmental pollutants into the host, implying that the gut microbiota will inevitably be affected [30]. However, there is currently a lack of research on the impact of cadmium exposure on the gut microbiota of ducks. In this study, we investigated the effects of environmental cadmium exposure on the gut microbiota of ducks.

## 2. Materials and Methods

### 2.1. Animals and Sample Acquisition

In this study, a total of 60 healthy one-day-old male Peking ducks were purchased from a commercial feedlot (Zhengzhou, China). These ducks were housed in the standard temperature, sanitary conditions, and illumination based on a previous study [31]. After acclimatization for seven days, these ducks were randomly assigned into two groups (30 ducks per group), namely, a control group (CON) and Cd exposure group (CAD). The dose of Cd was determined according to previous studies [28]. Cadmium sulfate (3CdSO_4_·8H_2_O) purchased from Shanghai Aladdin Biochemical Technology Co., Ltd., China, was used as the source of Cd. Throughout the trial, the ducks in both the control and Cd exposure groups were provided ad libitum access to water and food. However, the ducks in the experimental group required an additional supplementation of 4 mg/kg Cd in feed to induce Cd poisoning [28]. After 60 consecutive days, the ducks in both groups were euthanized by injecting pentobarbital (25 mg/kg). Cecal contents were snap-frozen in liquid nitrogen and stored at −80 °C for amplicon sequencing.

### 2.2. 16S rDNA Amplicon Sequencing

The frozen cecal contents were thawed and DNA was extracted following previous research [1]. A quality assessment was then conducted to ensure a qualified product. The primers (338F: ACTCCTACGGGAGGCAGCA and 806R: GGACTACHVGGGTWTCTAAT) were synthesized based on conserved regions of the 16S rRNA gene and used to amplify the V3/V4 regions. The PCR amplification procedure and conditions were based on previous reports [1]. The PCR products underwent further processing, including target-fragment recovery, agarose-gel-electrophoresis detection, and fluorescence quantification, to purify the amplification product and construct a sequencing library. Prior to sequencing, quality detection and fluorescence quantification were performed to check library quality, ensuring that the qualified library concentration was greater than 2 nM and had only one peak. Finally, the qualified library was gradiently diluted and denatured into single strands for paired-end sequencing using the MiSeq sequencing machine.

### 2.3. Bioinformatics and Data Analysis

The raw data generated by amplicon sequencing were quality assessed to eliminate unqualified sequences, including short sequences and chimeras, in order to obtain high-quality sequences. These high-quality sequences were then clustered and divided into OTUs. Taxonomic analysis was performed based on the operational-taxonomic-unit (OTU) analysis results to determine the microbial composition and abundance distribution at different taxonomic levels for each sample. Species distribution histograms and clustering heat maps were also created. To assess sequencing depth and evenness, rarefaction curves and rank abundance curves were generated. ACE, Chao1, Shannon, and Simpson indices were calculated based on the abundance of OTUs in each sample to evaluate the impact of cadmium exposure on the alpha diversity of gut microbiota in ducks. In addition, we generated PCOA diagrams to analyze the similarities and differences in the main components of gut microbiota among different groups. To investigate the impact of cadmium exposure on the composition of gut microbiota in ducks, we used LEfSe and Metastats analyses to identify differential taxa at various taxonomic levels. SPSS version 19.0 (SPSS Inc., Chicago, IL, USA) and GraphPad Prism 8.01 (GraphPadInc, La Jolla, CA, USA) were used to perform statistical analysis. The *p*-values (mean ± SD) less than 0.05 were considered statistically significant.

## 3. Results

### 3.1. Data Acquisition and Analysis

**Figure 1:**
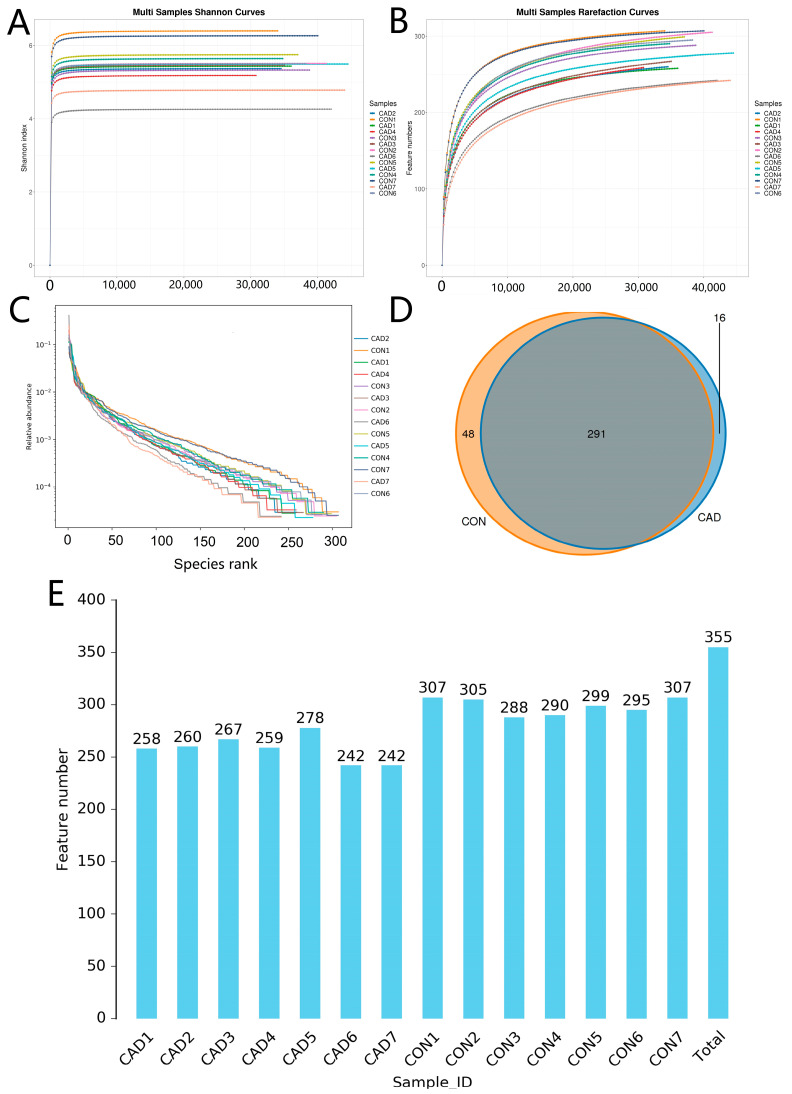
Sequencing depth assessment and OTU number statistics. (**A**) Shannon index curve. (**B**) Rarefaction curve. (**C**) Rank abundance curve. (**D**) The number of shared and individual OTUs between CON and CAD groups. (**E**) The number of OTUs in each sample.

Results indicated that a total of 787,328 (CON = 412,594, CAD = 374,734) raw reads were acquired from the CON and CAD groups, with an average of 56,237 reads per sample (varying from 43,186 to 64,590) (Table 1). After quality control, there are 567,312 (CON = 289,639, CAD = 277,673) high-quality reads in both groups, and its effective rate is approximately 72.06%. All the curves representing the sequencing depth of samples were saturated, indicating that almost all bacterial species could be covered (Figure 1A,B). Furthermore, we also observed that the rank abundance curves of each sample were flat, suggesting the sufficient evenness (Figure 1C). Following 97% of species similarity, these effective sequences from the CON and CAD groups were clustered into 355 (CON = 339, CAD = 307) OTUs, ranging from 242 to 307 OTUs per sample (Figure 1D,E). Among them, 291 OTUs are identified as common, accounting for approximately 81.97% of the total OTU quantity. Additionally, we also found 48 and 16 unique OTUs in the CON and CAD groups, which made up about 13.52% and 4.51% of the OTU composition, respectively.

### 3.2. Alterations in the Diversity Index Associated with Cadmium Exposure

**Figure 2:**
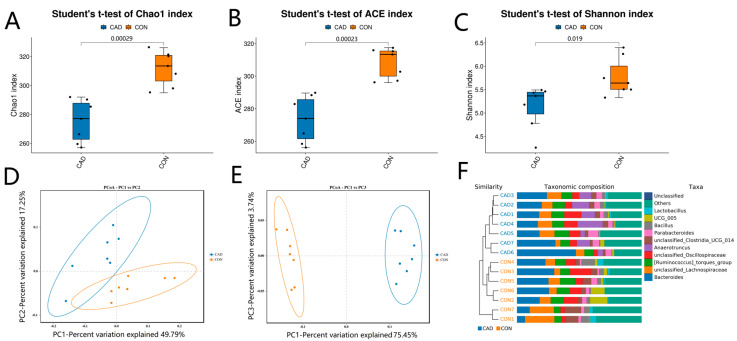
Effects of cadmium exposure on gut microbial diversity of ducks. The richness of gut microbiota is measured using Chao1 (**A**) and ACE (**B**) indices, whereas its diversity is measured using Shannon (**C**) index. The beta diversity of the gut microbiota is assessed through the analysis of weighted UniFrac PCoA plots (**D**), unweighted UniFrac PCoA plots (**E**), and UPGMA clustering trees (**F**).

To explore the negative effects of cadmium poisoning on gut microbiota in ducks, we computed and compared the diversity indices of the CON and CAD groups. Good’s coverage estimates in the samples of the CON and CAD groups varied from 99.89% to 99.97%, suggesting that almost all bacteria could be covered. Results of inter-group comparative analysis revealed that there were statistically significant differences in the ACE (308.24 ± 3.51 versus 273.43 ± 5.30, *p* = 0.00023), Chao1 (311.80 ± 4.53 versus 275.30 ± 5.51, *p* = 0.00029), and Shannon (5.77 ± 0.15 versus 5.14 ± 0.17, *p* = 0.019) indices between the CON and CAD groups, suggesting that cadmium exposure memorably reduced the diversity and abundance of gut microbiota in ducks (Figure 2A–C). Furthermore, PCoA maps representing the beta diversity were generated to further assess the changes of the main component of gut microbiota during cadmium exposure (Figure 2D,E). Results indicated that the samples in the CON or CAD groups were clustered together, showing a similar main component within the groups. However, the samples in the CON and CAD groups were clearly separated, demonstrating that the main component of gut microbiota was dramatically affected by the cadmium exposure. Moreover, UPGMA analysis results showed that samples from the same group were clustered together and separated from another group, indicating differences in the main components of the gut microbiota (Figure 2F).

### 3.3. Alterations in the Gut Microbial Composition after Cadmium Exposure

**Figure 3:**
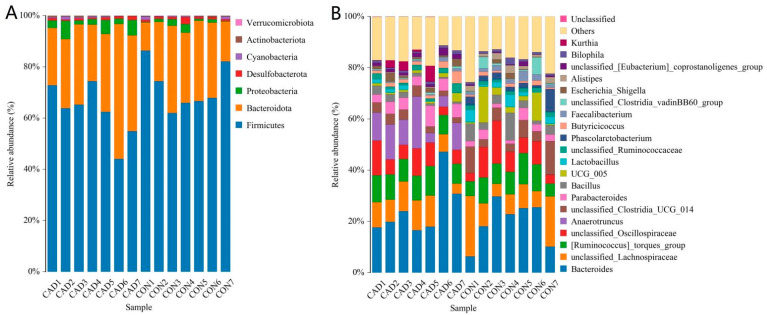
Effects of cadmium exposure on gut microbiota composition at phylum (**A**) and genus (**B**) levels. The *x*-axis represents the sample name, while the *y*-axis represents the relative abundance percentage. The histogram’s area represents the proportion of bacterial relative abundance.

**Figure 4:**
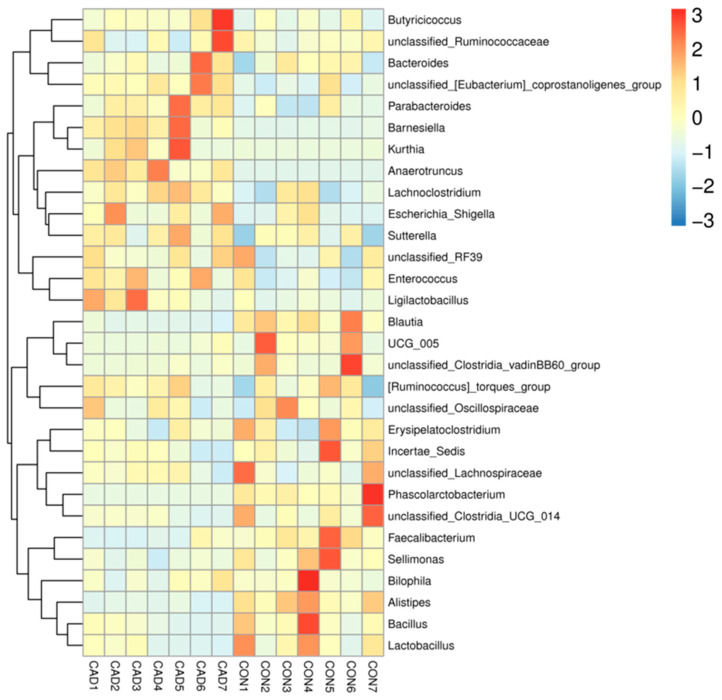
The clustered heatmap is utilized to visually display similarities and differences in the gut microbial composition of samples. The color gradient, ranging from blue to red, represents the abundance level across samples, with blue indicating low abundance and red indicating high abundance.

There were 7 phyla and 129 genera recognized from both groups, and the species and abundance of major bacterial phyla and genera are shown in Table 2. The phyla *Firmicutes* (72.20%, 61.69%), *Bacteroidota* (24.61%, 32.65%), and *Proteobacteria* (1.37%, 4.00%) were abundantly present in the CON and CAD groups, accounting for approximately 98% of the taxonomic groups identified (Figure 3A). Other phyla, such as *Desulfobacterota* (1.08%, 0.92%), *Cyanobacteria* (0.46%, 0.36%), *Actinobacteriota* (0.22%, 0.35%), and *Verrucomicrobiota* (0.04%, 0.00%), were represented with lower abundances. At the genus level, *Bacteroides* (25.52%), *Anaerotruncus* (9.85%), [*Ruminococcus*]*_torques_group* (9.35%), and *unclassified_Lachnospiraceae* (9.05%) were the four most predominant bacteria in the CAD group, accounting for over 53% of the total composition (Figure 3B). Furthermore, the dominant genera found in the CON group were *Bacteroides* (19.83%), *unclassified_Lachnospiraceae* (11.29%), [*Ruminococcus*]*_torques_group* (8.64%), and *unclassified_Oscillospiraceae* (8.46%), in descending order. Moreover, the effects of cadmium exposure on gut microbiota in ducks can also be shown by a clustering heatmap (Figure 4).

**Figure 5:**
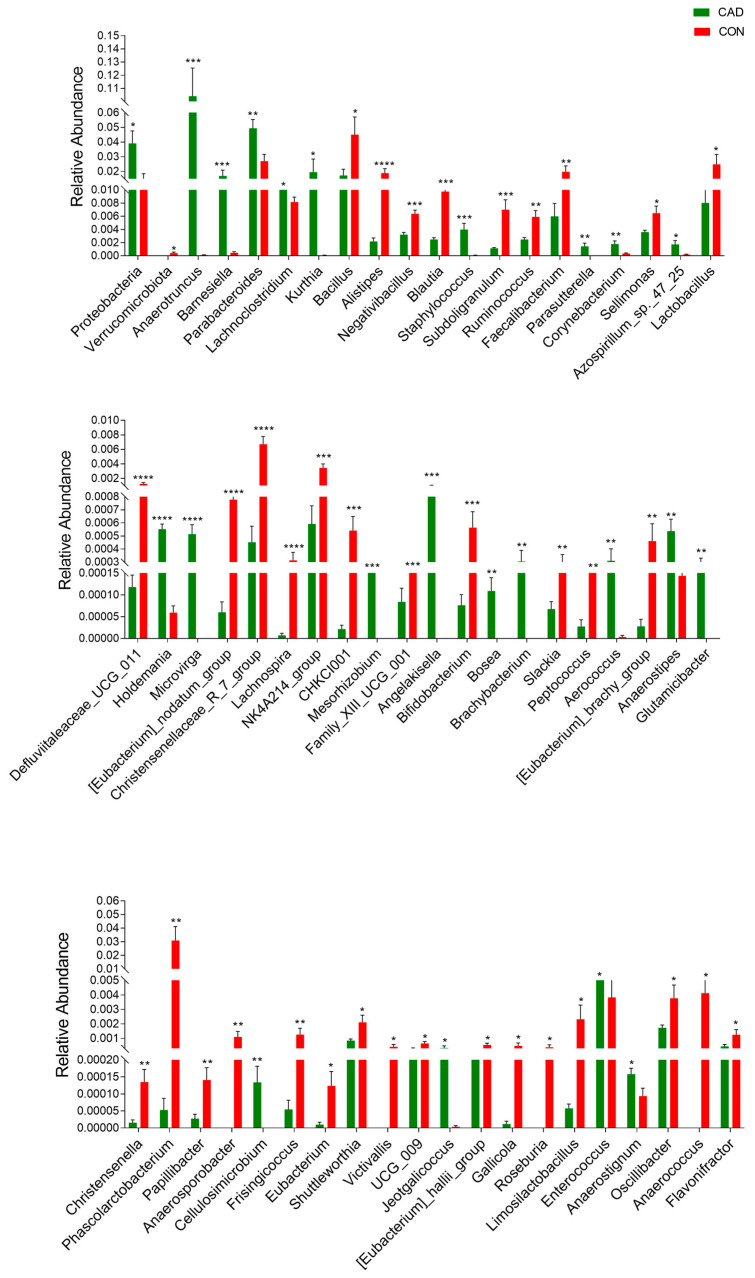
Statistics of significantly different bacteria between CON and CAD groups. The relative abundance of bacteria is represented by the area of the histogram. * *p* < 0.05, ** *p* < 0.01, *** *p* < 0.001, **** *p* < 0.0001.

**Figure 6:**
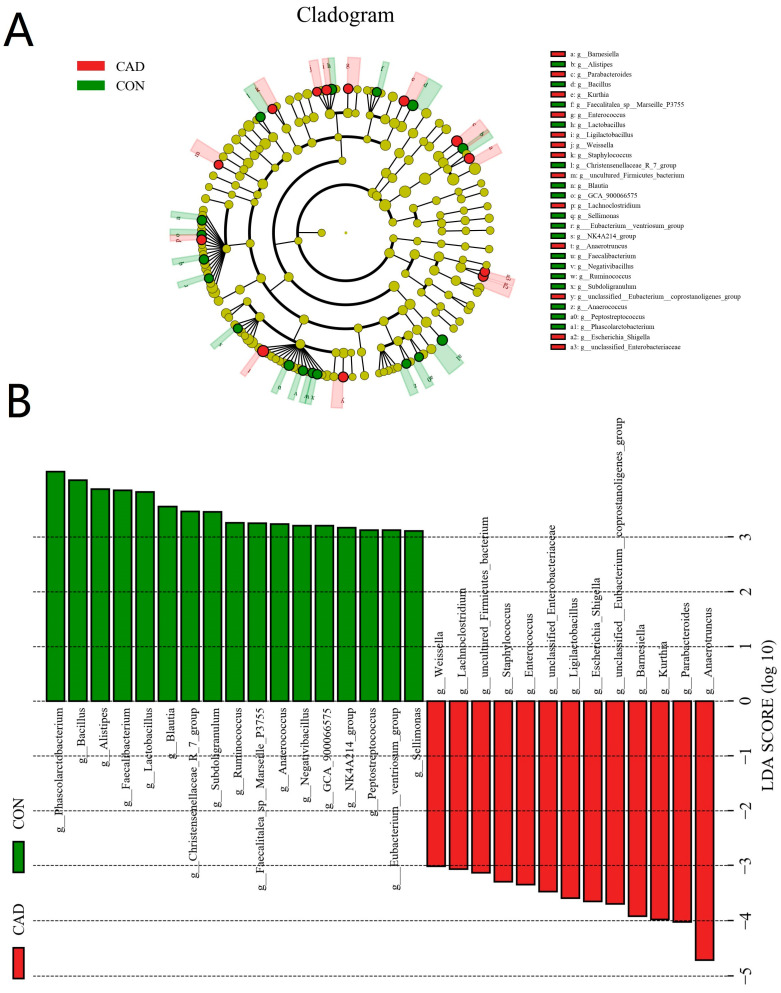
LEfSe analysis identifies bacteria that change significantly in the gut microbiota of ducks at different taxonomic levels during cadmium exposure. (**A**) The cladogram of bacteria at different taxonomic levels. (**B**) Histogram of linear-discriminant-analysis (LDA) value distribution. The ordinate indicates the taxa with significant differences between groups, and the abscissa visually displays the logarithmic score of LDA of each taxon. The longer the column, the more significant the difference between the two groups of this bacteria.

A Metastats analysis was used to distinguish differential taxa associated with cadmium exposure (Figure 5). The results demonstrated that cadmium exposure led to a notable increase in the level of *Proteobacteria*, while the level of *Verrucomicrobiota* exhibited a decrease. Moreover, there were also 58 bacterial genera that were dramatically different between the CON and CAD groups. Among them, the levels of 36 genera (*Bacillus*, *Alistipes*, *Negativibacillus*, *Blautia*, *Subdoligranulum*, *Ruminococcus*, *Faecalibacterium*, *Sellimonas*, *Lactobacillus*, *Defluviitaleaceae_UCG_011*, [*Eubacterium*]*_nodatum_group*, *Christensenellaceae_R_7_group*, *Lachnospira*, *NK4A214_group*, *CHKCI001*, *Family_XIII_UCG_001*, *Bifidobacterium*, *Slackia*, *Peptococcus*, [*Eubacterium*]_*brachy_group*, *Christensenella*, *Phascolarctobacterium*, *Papillibacter*, *Anaerosporobacter*, *Frisingicoccus*, *Eubacterium*, *Shuttleworthia*, *Victivallis*, *UCG_009*, *[Eubacterium]_hallii_group*, *Gallicola*, *Roseburia*, *Limosilactobacillus*, *Oscillibacter*, *Anaerococcus,* and *Flavonifractor*) dramatically decreased, whereas the relative proportions of 22 genera (*Staphylococcus*, *Anaerotruncus*, *Barnesiella*, *Parabacteroides*, *Lachnoclostridium*, *Kurthia*, *Parasutterella*, *Corynebacterium*, *Azospirillum_sp._47_25*, *Holdemania*, *Microvirga*, *Mesorhizobium*, *Angelakisella*, *Aerococcus*, *Brachybacterium*, *Anaerostipes*, *Glutamicibacter*, *Bosea*, *Cellulosimicrobium*, *Jeotgalicoccus*, *Enterococcus,* and *Anaerostignum*) dramatically increased during cadmium exposure. In addition to this discriminant analysis, we also performed LEfSe analysis to identify the differential bacteria between the CON and CAD groups (Figure 6). At the genus level, the CON group showed significantly higher abundances of *Eubacterium__ventriosum_group*, *Peptostreptococcus*, *GCA_900066575,* and *Faecalitalea_sp__Marseille_P3755*, while the CAD group was observably enriched for *unclassified__Eubacterium__coprostanoligenes_group*, *Escherichia_Shigella*, *Ligilactobacillus*, *unclassified_Enterobacteriaceae,* and *uncultured_Firmicutes_bacterium*.

### 3.4. Correlation Network Analysis

**Figure 7:**
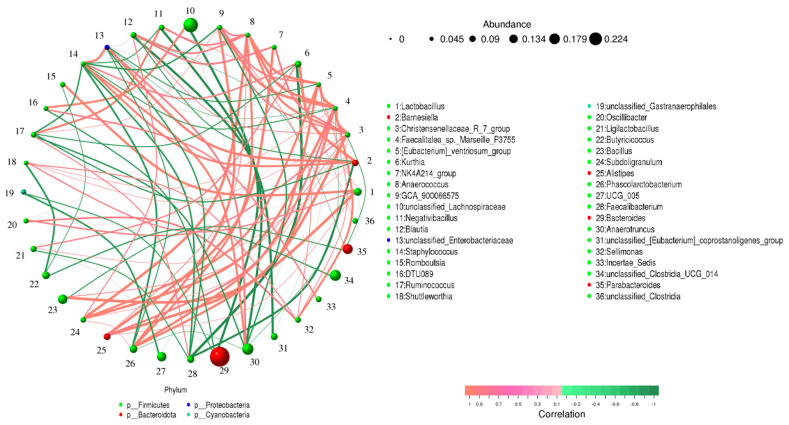
A visual network diagram depicting bacterial correlations was created by considering the abundance and variation of bacteria in each sample. The lines in the diagram represent correlations between bacteria, with negative correlations shown in green and positive correlations shown in red.

*Lactobacillus* was positively related to *Bacillus* (0.93) but negatively associated with *Parabacteroides* (−0.76) (Figure 7). *Christensenellaceae_R_7_group* was positively associated with *Alistipes* (0.93), *Bacillus* (0.79), *Blautia* (0.78), *Subdoligranulum* (0.78), and *Lactobacillus* (0.78) but negatively related to *Parabacteroides* (−0.79). *Ruminococcus* was positively associated with *Negativibacillus* (0.85) and *Faecalibacterium* (0.80) but negatively related to *Anaerotruncus* (−0.82), *Barnesiella* (−0.81), and *Kurthia* (−0.80). *Blautia* was negatively associated with *Barnesiella* (−0.80) and *Parabacteroides* (−0.78). *Oscillibacter* was positively related to *Christensenellaceae_R_7_group* (0.81) and *Sellimonas* (0.82).

## 4. Discussion

Cadmium, a prevalent and persistent environmental contaminant, poses significant biological toxicity, such as teratogenic, carcinogenic, and mutagenic effects [32]. It remains present in water, soil, and sediment and can easily migrate, endangering ecosystems and food safety [33]. Animals could be exposed to cadmium-contaminated food, water, and dust through various pathways, causing organ dysfunction and structural changes [34,35]. Numerous studies have demonstrated that chronic exposure to heavy metals, like cadmium, can indeed induce oxidative stress reactions and inflammation, leading to various forms of cellular and tissue damage [36,37]. Orisakwe et al. conducted a study that revealed significantly elevated cadmium concentrations in poultry bones from lead-contaminated gold mines, accompanied by severe bone metabolic disease [38]. Waterfowl, including ducks, are more likely to be exposed to cadmium-contaminated water and feed compared with other animals. Previous studies have extensively examined the effects of cadmium exposure on duck kidneys and bones [19,29]. However, there is a gap in knowledge regarding the impact of cadmium exposure on the gut microbiota of ducks. Therefore, the objective of our study was to investigate the effects of cadmium exposure on gut microbiota homeostasis in ducks.

Chao1 and ACE are commonly used to assess the abundance of gut microbiota, while Simpson and Shannon are used to assess its diversity [39]. Therefore, diversity indices, such as Chao1, ACE, Simpson, and Shannon, are important indicators for evaluating the homeostasis of gut microbiota. Previous research has shown that heavy metals can contaminate the surrounding environment through several channels, such as discharge, rainwater runoff, and infiltration [40,41]. These heavy metals can enter the host through the food chain, posing a risk to the host’s health [21]. Since the intestine is the main pathway for food intake, digestion, and absorption, it is inevitably affected by heavy metals present in food [42]. For instance, Yang et al. demonstrated that cadmium exposure in adolescent rats can result in gut microbiota and metabolic disturbances, along with chronic inflammation and dysfunction in multiple organs [43]. Similarly, Ba et al. found that cadmium exposure can reduce the diversity and main components of the gut microbiota, thereby affecting liver lipid metabolism [44]. However, research on the toxicity of cadmium in poultry has primarily concentrated on kidney and bone damage, with limited investigation into its effects on gut microbiota. Consistent with previous studies, this study also observed that cadmium exposure can decrease the gut microbial Chao1, ACE, Simpson, and Shannon indices of duck, resulting in gut microbial dysbiosis. Furthermore, cadmium was found to significantly alter the major components of gut microbiota in ducks. Despite similar diets and habitats between the control and experimental ducks, we observed significant differences in the alpha and beta diversity of their gut microbiota. These findings suggest that cadmium may be an important driving force leading to changes in gut microbiota.

This study indicated that *Firmicutes*, *Bacteroidota,* and *Proteobacteria* were the most predominant bacterial phyla in the CON and CAD groups, which were not affected by cadmium. Furthermore, these bacterial phyla were also abundant in other poultry species, indicating that this may be a dominant feature of gut microbiota in poultry [45,46]. Although the types of the predominant bacterial phyla did not alter, their abundances, such as for *Proteobacteria,* increased significantly during cadmium exposure. *Proteobacteria* is the largest bacterial phylum consisting of some Gram-negative bacteria. Early investigations have shown that some members of the *Proteobacteria* are pathogenic or opportunistic pathogens that may cause many gastrointestinal diseases and seriously threaten the health of the host [47,48]. Therefore, a higher abundance of *Proteobacteria* in the intestine may induce host immune responses, increasing the risk of pathogen infection. Previous studies demonstrated that heavy metal could perturb gut microbial homeostasis through changing bacterial compositions and abundances [16,49].

In this investigation, we also found significant changes in the gut microbiota of ducks exposed to cadmium. Notably, several quantitatively reduced bacterial genera, such as *Christensenellaceae*, *Bifidobacterium*, *Ruminococcus*, *Oscillibacter*, *Roseburia*, *Blautia*, *Alistipes*, *Bifidobacterium*, *Peptococcus*, *Faecalibacterium*, *Roseburia*, and *Lactobacillus,* were regarded as beneficial bacteria. *Christensenellaceae* was shown to have the ability to produce hydrolytic enzymes associated with feed efficiency, indicating an important role in growth performance [50]. Additionally, it participates in the active regulation of the intestinal environment and is closely related to host health homeostasis and immune regulation [51]. As recognized intestinal beneficial bacterium, *Bifidobacterium* is widely used in food, medicine, and feed production due to its multiple health benefits to the host. For instance, investigations have shown that *Bifidobacterium* plays an important role in maintaining intestinal microbial homeostasis, improving the intestinal environment and preventing pathogenic bacterial infections [52,53]. Moreover, *Bifidobacterium* can also promote the absorption and utilization of minerals and stimulate the intestinal peristalsis and immune system [54,55]. In addition to the above properties, it also contributes to relieving and improving various gastrointestinal diseases, such as diarrhea, constipation, and enteritis [56,57]. *Ruminococcus* has been reported to degrade cellulose and starch [58]. It has been reported that the abundance of *Peptococcus* in the intestine is strongly positively correlated with the growth performance of the host [59]. As one of the most abundant and important commensal bacteria, *Faecalibacterium* has been shown to produce butyrate, relieve inflammation, and immunomodulate [60]. Some studies found that the abundance of *Faecalibacterium* showed a downward trend in patients with Crohn’s disease and ulcerative colitis [61]. *Roseburia* has been shown to have the ability to utilize sugars and produce butyrate [62]. *Lactobacillus* has been considered one of the most important intestinal beneficial bacteria due to its positive regulatory effect on the intestinal barrier and mucosal immunity [63,64]. Moreover, *Lactobacillus* has also been reported to improve host growth performance, antioxidant capacity, and digestive enzyme activity [63,65]. In addition to maintaining gut microbiota and antibacterial effects, it also plays an important role in lowering cholesterol and preventing and relieving diarrhea and intestinal inflammation [66]. Notably, these decreased-abundance bacteria, including *Ruminococcus*, *Oscillibacter*, *Roseburia*, *Blautia*, *Alistipes,* and *Bifidobacterium*, were identified as potential producers of short-chain fatty acids (SCFAs). SCFAs are considered to be a class of intestinal metabolites that are beneficial to host health. Previous studies have shown that SCFAs help to reduce oxidative stress, ease intestinal inflammation, and maintain intestinal permeability [67]. Furthermore, studies have reported that they can inhibit the invasion of pathogenic bacteria and maintain the gut microbial balance [68]. Recent studies involving SCFAs have also revealed their important roles in host immunity, metabolism, and disease prevention [69]. Consistent with the current study, there have also been some previous reports demonstrating that heavy-metal exposure reduces the number of SCFA-producing bacteria in the intestine [50]. In this research, we also observed that cadmium exposure led to a distinct increase in several conditioned and pathogenic bacteria, including *Jeotgalicoccus*, *Staphylococcus*, *Enterococcus*, *Brachybacterium*, *Aerococcus*, *Parasutterella,* and *Corynebacterium*. As a common resistant bacterium, *Jeotgalicoccus* was previously demonstrated to be inversely associated with poultry productivity. Previous studies have shown that *Staphylococcus* infection was strongly associated with the development of several diseases, such as septic sepsis, meningitis, and pneumonia [70,71]. Furthermore, it can also cause host diarrhea, gastroenteritis, vomiting, and fever by producing staphylolysin, coagulase, and enterotoxin [72,73]. *Brachybacterium* and *Aerococcus* have been demonstrated to cause bloodstream infection and endocarditis, respectively [74,75]. *Parasutterella* has been shown to play an important role in the development of Crohn’s disease, chronic intestinal inflammation, and irritable bowel syndrome [76]. As a common pathogen, *Corynebacterium* can cause lung abscess, bacteremia, and caseous lymphadenitis [77]. Previous studies have shown that *Enterococcus* can cause sepsis, pericarditis, and meningitis [78,79]. Furthermore, *Enterococcus* infections are difficult to treat with antibiotics due to inherent and acquired resistance [80]. The above-mentioned bacteria played vital roles in host health and intestinal functions. Thus, we suspected that cadmium may further induce the dysbiosis of gut microbiota via changing these functional bacteria. Research has demonstrated that bacteria are capable of interacting in various ways to uphold gut microbial homeostasis [30]. Consequently, changes in certain bacteria can potentially influence the functioning of other bacteria. In our study, we observed noteworthy correlations among certain bacteria, which could play a vital role in maintaining intestinal homeostasis. These findings suggest that cadmium can indirectly alter certain bacteria through intermicrobial interactions, thereby amplifying its impact on gut microbiota.

## 5. Conclusions

In summary, this research investigated the negative effects of cadmium exposure on gut microbial homeostasis in ducks. Results showed that cadmium exposure dramatically decreased gut microbial abundance and diversity, accompanied by significant variations in the main component of gut microbiota. This research provides novel insights into the toxic mechanism of cadmium exposure from the perspective of gut microbiota. Furthermore, these findings are also beneficial to increase public concern regarding the health threat caused by cadmium pollution, which may further provide motivation for regulating metal waste emission to ensure animal health and environmental quality.

## Figures and Tables

**Table 1:** Statistics of sequences generated during amplicon sequencing. GC(%) represents the GC content of the sample, which indicates the proportion of G- and C-type bases in the total number of bases. Q20(%) indicates the percentage of bases with a quality value of 20 or higher in the total bases. Similarly, Q30(%) represents the percentage of bases with a quality value of 30 or higher in the total bases.

Sample	Raw Reads	Clean Reads	Effective Reads	AvgLen (bp)	GC (%)	Q20 (%)	Q30 (%)	Effective (%)
CAD1	49,376	37,217	37,079	413	51.91	99.94	99.52	75.1
CAD2	47,727	35,427	35,348	415	51.84	99.93	99.48	74.06
CAD3	48,680	36,216	36,024	415	51.43	99.93	99.47	74
CAD4	43,186	32,376	31,971	411	52.16	99.93	99.5	74.03
CAD5	64,590	48,332	47,062	415	51.65	99.94	99.51	72.86
CAD6	62,329	48,171	45,133	417	50.25	99.93	99.48	72.41
CAD7	58,846	45,378	45,056	415	51.38	99.94	99.52	76.57
CON1	62,327	45,367	41,099	412	52.2	99.93	99.5	65.94
CON2	57,390	42,638	42,318	412	52.15	99.94	99.52	73.74
CON3	58,440	44,221	41,845	415	51.33	99.93	99.5	71.6
CON4	58,091	43,488	39,230	416	51.95	99.93	99.48	67.53
CON5	58,144	44,068	40,895	414	51.33	99.94	99.53	70.33
CON6	53,779	39,464	39,239	413	51.61	99.93	99.45	72.96
CON7	64,423	47,898	45,013	413	51.89	99.93	99.49	69.87

**Table 2:** The quantity of bacterial species at different taxonomic levels per sample.

Sample	Phylum	Class	Order	Family	Genus
CAD1	6	9	27	52	102
CAD2	6	10	28	52	105
CAD3	6	11	28	54	110
CAD4	6	10	28	53	103
CAD5	6	10	28	55	110
CAD6	6	10	29	55	108
CAD7	6	10	27	51	104
CON1	7	12	29	52	111
CON2	7	12	29	51	106
CON3	7	12	29	49	105
CON4	7	12	28	48	105
CON5	7	12	29	51	107
CON6	7	12	29	48	103
CON7	7	12	29	53	113
Total	7	12	31	61	129

## Data Availability

The original sequence data were submitted to the Sequence Read Archive (SRA) (NCBI, Bethesda, MD, USA) with the accession No. PRJNA1010493.
